# Integrative Radiogenomics Approach for Risk Assessment of Postoperative and Adjuvant Chemotherapy Benefits for Gastric Cancer Patients

**DOI:** 10.3389/fonc.2021.755271

**Published:** 2021-11-05

**Authors:** Yin Jin, Yilun Xu, Yanyan Li, Renpin Chen, Weiyang Cai

**Affiliations:** ^1^Department of Gastroenterology, The First Affiliated Hospital of Wenzhou Medical University, Wenzhou, China; ^2^Department of Urology, Second Affiliated Hospital & Yuying Children’s Hospital of Wenzhou Medical University, Wenzhou, China

**Keywords:** radiogenomics, adjuvant chemotherapy benefit, postoperative gastric cancer, nomogram, survival prediction

## Abstract

Gastric cancer (GC) is a typical heterogeneous malignant tumor, whose insensitivity to chemotherapy is a common cause of tumor recurrence and metastasis. There is no doubt regarding the effectiveness of adjuvant chemotherapy (ACT) for GC, but the population for whom it is indicated and the selection of specific options remain the focus of present research. The conventional pathological TNM prediction focuses on cancer cells to predict prognosis, while they do not provide sufficient prediction. Enhanced computed tomography (CT) scanning is a validated tool that assesses the involvement of careful identification of the tumor, lymph node involvement, and metastatic spread. Using the radiomics approach, we selected the least absolute shrinkage and selection operator (LASSO) Cox regression model to build a radiomics signature for predicting the overall survival (OS) and disease-free survival (DFS) of patients with complete postoperative gastric cancer and further identifying candidate benefits from ACT. The radiomics trait-associated genes captured clinically relevant molecular pathways and potential chemotherapeutic drug metabolism mechanisms. Our results of precise surrogates using radiogenomics can lead to additional benefit from adjuvant chemotherapy and then survival prediction in postoperative GC patients.

## Introduction

Gastric cancer (GC) is now the third most common cancer and the second leading cause of cancer-related mortality worldwide (1), of which nearly three-quarters occurred in Asia, and more than two-fifths occurred in China (2). Pathological staging according to the TNM (tumor, lymph node, and metastasis) system and histological subtype have been commonly recognized as the most used master for the prognostic definition and treatment strategy choice in GC. Complete surgical excision is generally conducted as the primary intervention for the majority of stage I–III and partial stage IV GC patients, but the 5-year recurrence and survival rate still spread over a broad range. These findings reflected that GC is characteristic of biological heterogeneity with large variations in clinical outcomes even among those with the same stage. Thus, it is urgent to improve the prediction of GC prognosis by developing a novel signature to categorize patients and predict further survival.

Radiomics is a newly developing approach that transfers imaging data into a high-dimensional mineable feature space using a large number of automatically applied data-characterization algorithms (3, 4). Radiomics translates the genomic heterogeneity into expression in an intratumoral heterogeneity through imaging (5, 6). On the other hand, radiomics signature has the power to capture intratumoral heterogeneity through a noninvasive method. Previous studies have demonstrated that the prognosis and malignant degree of GC were closely related to imaging features. For example, metabolically active tumor volume (MATV) has been proven to be a prognostic factor in patients with GC (7); Li et al. constructed a radiomics signature of 18-F fluorodeoxyglucose PET/CT for prediction of GC survival (8); Jiang et al. selected 19 potential predictors from the 269 features identified, which provided a neoteric angle for individualized diagnosis and prediction of malignancy potential for GC patients (9); Jiang et al. developed machine learning for predicting the pathological stage for GC (8); and studies established a deep learning radiomics model for effectively predicting the lymph node metastasis of local GC (10). However, these radiomics studies did not show satisfied diagnostic efficiency, and many controversial results still existed. The principal underlying explanations might be that these previous studies were only based on dated imaging technology, which only extracted 269 features from the non-filtered segmented ROI, and that the results were easily influenced by different individuals and lacked a proper validation. More importantly, a radiomics nomogram research investigating the association of post-operative GC and candidates’ selection for ACT has not yet been fully reported. Thus, more contributing factors should be offered for choice in the intended population and ideal regimens before therapy selection.

In this study, we adopted a quantitative radiomics approach and developed a multiple-feature-based radiomics signature, which function in predicting survival and assessing benefit from ACT for postoperative GC patients. Additionally, we also firstly explored the potential biological basis of radiomics with imaging and gene expression data. The radiomics trait-associated genes captured clinically relevant molecular pathways and potential chemotherapeutic drug metabolism.

## Material and Methods

### Study Population and Design

**Figure 1:**
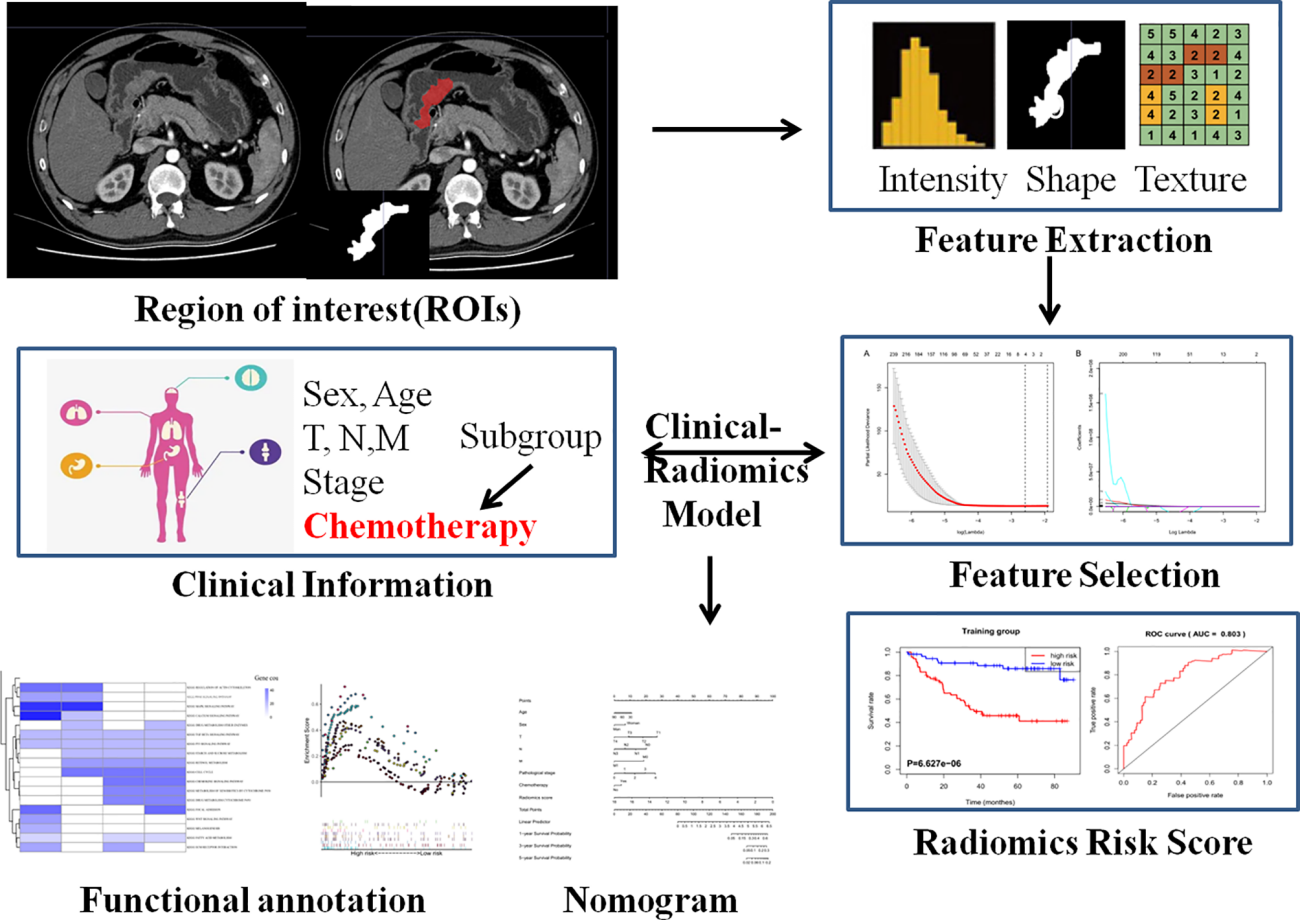
Flowchart of the study design and main process.

**Figure 1** presents the workflow of the study. We utilized two independent datasets in this study that were re-collected from an institution in China and from open-access online repositories, respectively. This study was approved by the institutional research board of the First Affiliated Hospital of Wenzhou Medical University. The records and images of 428 persons diagnosed with GC between January 2014 and January 2017 were reviewed. All these patients satisfied the following inclusion criteria: 18 years or over; firstly diagnosed with primary gastric cancer; excluded other malignant tumors; with CT images within 1 month prior to therapy; complete resection of the tumor tissue; without serious heart, lung, or kidney dysfunction; complete pathology and laboratory; and able to provide informed consent. Simultaneously, a part of the chosen patients successively underwent surgery and 8–12 regular periods of standard chemotherapy (including S1 alone, XELOX and FOLFIRI/FOLFOX). All of these patients were followed up, and recurrent and dead patients were recorded during the follow-up. The cutoff time of the study was set in May 2021. After exclusion, 417 enrolled patients were divided into two datasets: 172 patients were assigned to the training set, whereas 245 patients were assigned to the validation set. On the other hand, a dataset comprised CT imaging data and matched RNA sequencing data of 47 resected GC were obtained from the TCGA database of The Cancer Imaging Archive (TCIA) to evaluate the biological process of radiomics signature. Disease-free survival (DFS) was defined as the duration from the date of diagnose to that of recurrence, death, or the last follow-up. Overall survival (OS) was defined as the duration from diagnosis to death or the last follow-up.

### Feature Extraction and Selection

For each patient, the tumor region was contoured in a slice-by-slice manner on CT images by two experienced radiologists using MRIcroGL and 3D Slicer software (http://www.mccauslandcenter.sc.edu, www.slicer.org) (11, 12). We manually segmented the contour of the tumor in the arterial phase 5-mm CT images for image feature extraction. An open-source platform, PyRadiomics in Python (https://github.com/mvallieres/radiomics/), was utilized to extract 799 radiomics features from the selected segmented ROI (13), which include seven types of indexes, namely, Shape; First-Order Statistics; Gray Level Co-occurrence Matrix; Gray Level Run Length Matrix; Gray Level Size Zone Matrix; Neighboring Gray Tone Difference Matrix; and Gray Level Dependence Matrix. Feature selection is essential to avoiding overfitting, which was devised to reduce error to the high-dimensional radiomics features. At first, the intraclass correlation coefficients (ICCs) were calculated between the features extracted from the two radiologists, and features with either intra-observer or interobserver ICCs less than 0.75 were excluded. Secondly, features with significant differences between PD and non-PD (CR + PR + SD) groups were selected through a t-test (14).

### Construct Radiomics Score and Combination Nomogram

The least absolute shrinkage and selection operator (LASSO) method was applied to select the most optimal radiomics feature subsets for predicting the radiomics score. A radiomics score was calculated for each patient *via* weighting by their LASSO Cox coefficients: wavelet-LLH glszm SizeZone NonUniformity Normalized*1.8977 – wavelet-LHL glcm Cluster Shade*0.0009 + wavelet-LHH first order Kurtosis*0.005364 + wavelet-HLH glszm Large Area Low Gray Level Emphasis*5.97E-06. Setting the median score as the cutoff line, the GC cohort was divided into low- and high-risk groups, then K–M survival analysis was employed. Furthermore, this calculation and the cutoff value were applied to the validation cohort.

The OS and DFS nomograms were constructed based on the main prognostic factors to predict the 1-, 3-, and 5-year survival for patients. Each patient could sum up variable scores and finally establish predictive measures of survival and relapse. The calibration curve for predicting the 1-, 3-, and 5-year OS and DFS indicated that the nomogram-predicted survival closely corresponded with actual survival outcomes. The survival analysis was conducted using rms, survival, and survcomp package. Hazard ratios (HRs) and 95% confidence intervals (CIs) were recorded.

### Gene Set Enrichment Analysis and Functional Annotation

Gene set enrichment analysis (GSEA) was performed to explore the biological basis of the radiomics signature for prognosis prediction (15). The raw RNA-seq expression data were downloaded and normalized using the limma package. GO enrichment analysis of the signature genes was conducted using the R package clusterProfiler. Significantly enriched biological processes are summarized.

### Statistical Analysis

The experimental data were analyzed by Prism 5.0 software (GraphPad) and R software (version 3.4.2, http://www.R-project.org). Student’s t test and Wilcoxon’s test were utilized to compare continuous variables and ordered categorical variables. Survival analysis was performed using the Kaplan–Meier method, and the survival of the clusters was compared using the log-rank test.

## Results

### Baseline Clinic-Pathological Characteristics

At first, a total of 417 GC patients from Wenzhou Medical University met the inclusion criteria from January 1, 2014, to January 1, 2016. In this study, about 43.9% of the patients only received surgery, while the others conducted both adjuvant postoperative therapy and surgery. As of March of 2021, 183 patients died during follow-up and none were lost to follow-up. Among the total patients, 281 patients (60.0%) achieved tumor control (CR + PR + SD), while 136 patients (40.0%) had progressive disease. The median follow-up time was 30.25 and 31.42 months in the training and validation cohorts, respectively. There was no significant difference between the two cohorts in terms of clinicopathologic factors or follow-up time. The baseline clinico-pathological parameters are summarized in **Supplementary Table 1**.

### Radiomics Feature Selection and Signature Construction

**Figure 2:**
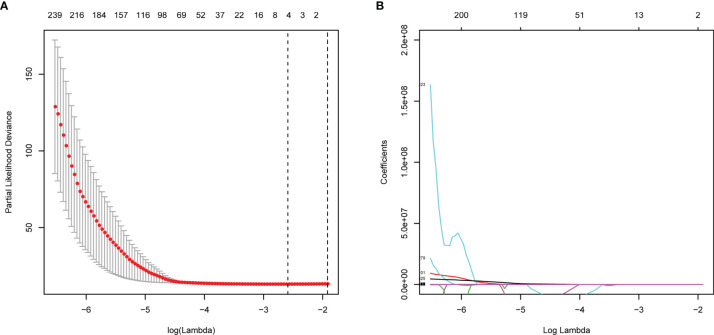
Feature selections with LASSO Cox regression analysis. **(A)** λ selection in the LASSO model using 10-fold cross-validation. **(B)** LASSO coefficient profiles of all the radiomics features. Vertical black dashed line represents the optimal resulted in nine nonzero features.

**Figure 3:**
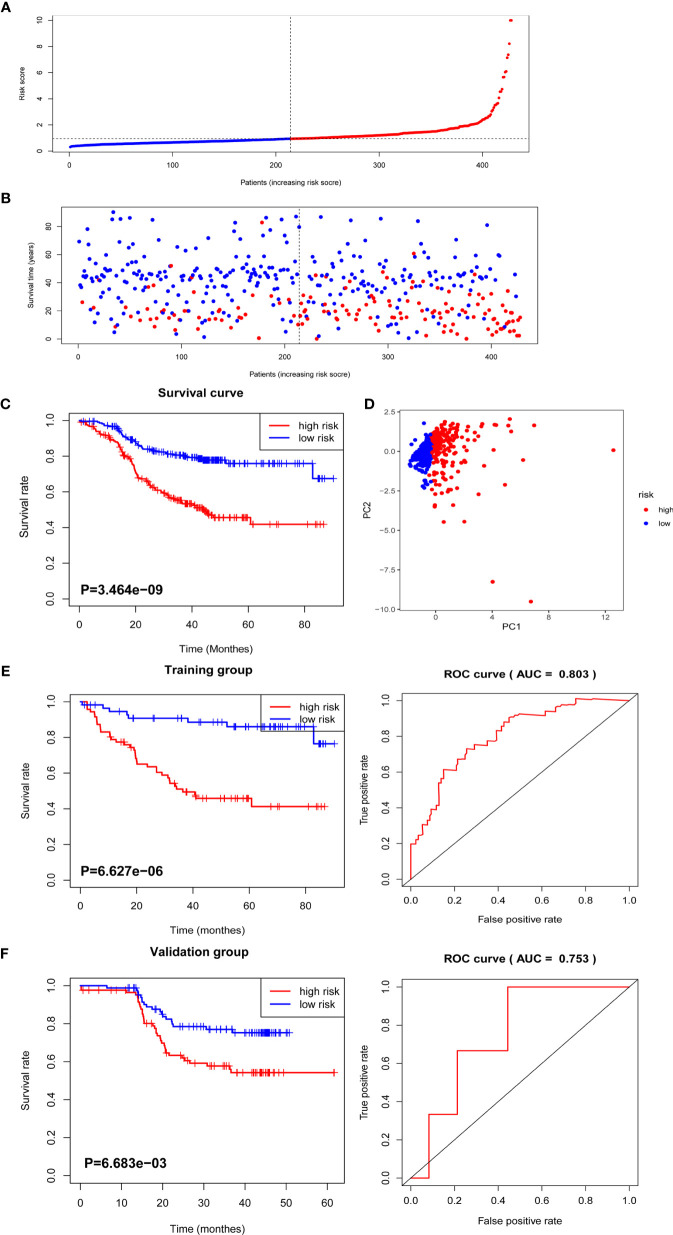
The distribution of radiomics score and overall survival analysis. **(A)** The distribution of the radiomics score of GC patients. **(B)** The overall survival status for each GC patient. **(C)** Overall survival curve of total GC patients. **(D)** PCA depicts the variation of high- and low-radiomics scores. **(E)** Overall survival and ROC curves of the training group. **(F)** Overall survival and ROC curves of the validation group.

A total of 799 features were extracted from the tumor volume, in which each sample quantified the intratumor heterogeneity by using the Gray Level Co-occurrence (GLCM), Gray Level Run Length Matrices (GLRLM), Gray Level Size Zone Matrix (GLSZM), Neighboring Gray Tone Difference Matrix (NGTDM), and Gray Level Dependence Matrix (GLDM) indexes. We firstly excluded redundant features with ICCs less than 0.75 and then conducted the t-test to select the features with statistically significant differences between PD and non-PD (CR + PR + SD) groups. Finally, four features were selected *via* LASSO regression (**Figure 2** and **Supplementary Figure 1**). The radiomics score of each GC case was calculated by a weighted linear combination of these four features and their corresponding coefficients. For low- and high-risk GC patients, the median radiomics score was selected as the cutoff line (**Figures 3A, B**). In either the total, training, or validation set, the median radiomics scores in the low-grade group were higher than those in the high-grade group (p < 0.001 for all patients, **Figure 3C**). We eliminated the interaction between the components of the original data by PCA analysis (**Figure 3D**). The overall survival distribution and ROC curves shown in **Figures 3E, F** yield significant prognosis outcome and AUCs of 0.803 for the training set and 0.753 for the validation set, demonstrating the discriminative power of the radiomics signature. Moreover, similar results were also obtained for the PFS analysis (**Supplementary Figure 2**).

To verify that the accuracy of the radiomics model was also important in additional GC cases, we further selected TCGA GC cohorts for validation. Consistent with the above results, the high radiomics subgroup had a worse prognosis than the low one. The distribution of radiomics score and survival information of patients were analyzed and are shown in **Supplementary Figures 3A, B**.

### ACT Benefit Analysis Based on the Radiomics Score

**Figure 4:**
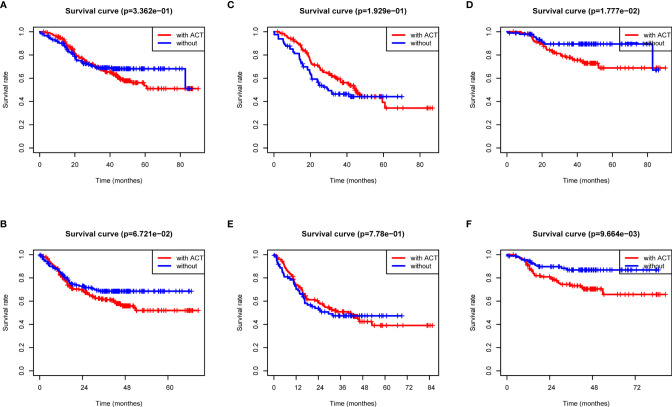
ACT benefit analysis based on the radiomics score. **(A)** Overall survival analysis of all GC patients according to ACT. **(B)** Disease-free survival analysis of all GC patients according to ACT. **(C)** Overall survival analysis of low-radiomics score GC patients according to ACT. **(D)** Overall survival analysis of high-radiomics score GC patients according to ACT. **(E)** Disease-free survival analysis of low-radiomics score GC patients according to ACT. **(F)** Disease-free survival analysis of high-radiomics score GC patients according to ACT.

Previous data suggested that image features are closely associated with chemotherapy efficacy; thus, we evaluated the benefit of chemotherapy according to the level of radiomics score in this study. As shown in **Figures 4A, B**, the adoption of ACT (n = 234/428) did not show significant OS and DFS survival benefit in all patients with complete postoperative GC patients (p = 0.3362 and 0.067, respectively). Using the median radiomics score as the cutoff line, we divided patients by chemotherapy therapy. As for the low radiomics subgroup, GC patients obtained a terrible response to ACT (**Figures 4C, D**), while patients with a high risk point showed no significant survival difference with or without ACT (**Figures 4E, F**). For patients with low radiomics scores, more effective systemic approaches to improve treatment outcomes need to be identified. Thus, the radiomics score was both a prognostic and predictive tool for post-operation GC patients.

### Radiomics Nomogram Construction

**Figure 5:**
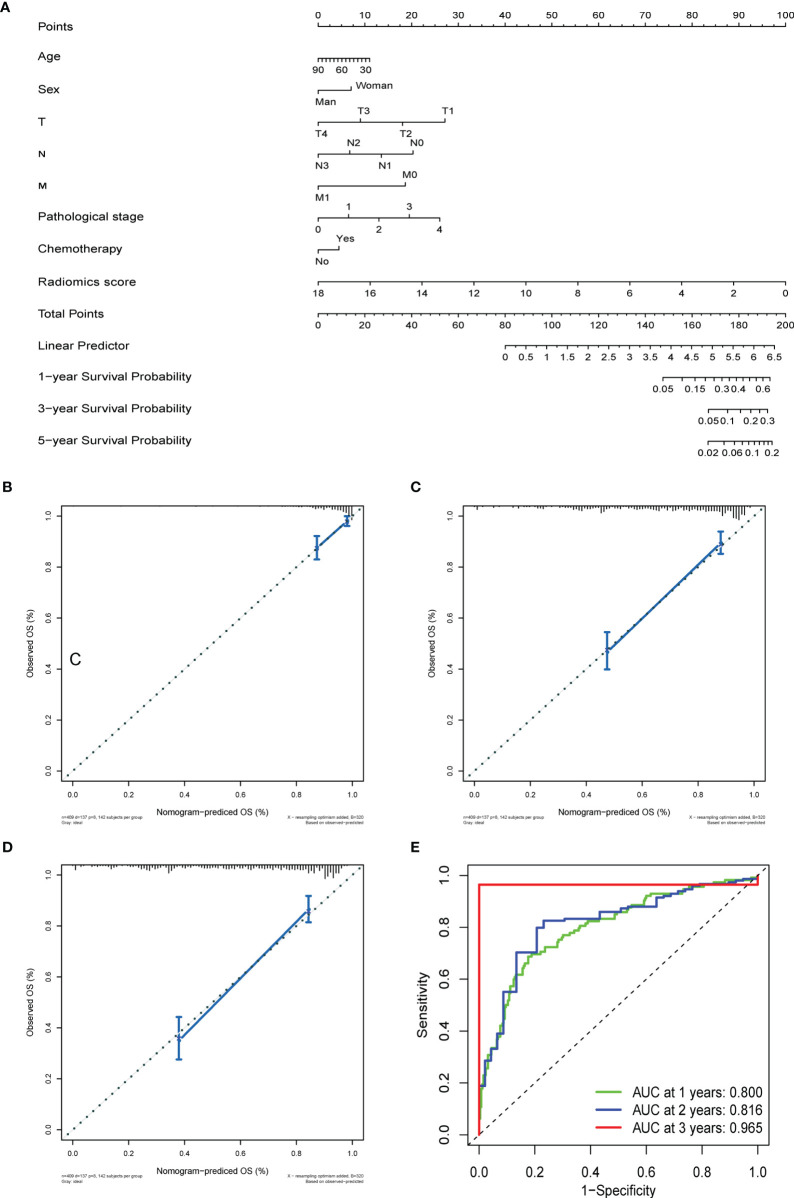
The prediction performance analysis of overall survival. **(A)** The nomogram for predicting 1-, 3-, and 5-year OS after surgery. **(B)** Calibration curve for risk of 1-year overall survival. **(C)** Calibration curve for risk of 3-year overall survival. **(D)** Calibration curve for risk of 5-year overall survival. **(E)** Receiver operating characteristic (ROC) curves for 1-, 3-, and 5- year overall survival.

For OS and DFS, the multivariate analysis demonstrated that glszm Size Zone NonUniformity Normalized, glcm Cluster Shade, First-Order Kurtosis, and glszm Large Area Low Gray Level Emphasis were all significantly associated with survive (p ≤ 0.05, **Supplementary Figure 1**). We further incorporated radiomics signature with other clinical factors, which has been proven to add prognostic information to better identify patients with different outcomes, and the radiomics nomogram was a good witness. As shown in **Figure 5**, the radiomics signature combined with TNM staging significantly reinforced the prognostic ability.

OS and DFS nomograms were constructed to predict the 1-, 3-, and 5-year overall survival and relapse of the GC (**Figure 5A** and **Supplementary Figure 4A**). Total score summations of each variable were based on the intersection of the vertical line. As shown in **Figure 5A**, radiomics score contributed the most risk points (ranged, 0–100), whereas other clinical information contributed much less (ranged, 0–40). By using this nomogram, we could convert each clinical index to the corresponding point and then calculate the total point, which was used to evaluate the 1-, 3-, and 5-year survival or relapse rate. Moreover, decision curve analysis showed a high accuracy of the predictive prognostic radiomics score (**Figures 5B–D**, and **Supplementary Figures 4B**, **D**). Decision curve analysis showed great predictive accuracy of prognostic nomograms for OS and DFS. In other words, the clinical-radiomics nomogram that incorporated both clinical risk factors and radiomics parameters showed excellent performance, with high 1-, 3-, and 5-year AUCs of 0.80, 0.816, and 0.965, respectively (**Figure 5E** and **Supplementary Figure 4E**).

### Biological Basis of the Radiomics Signature

**Figure 6:**
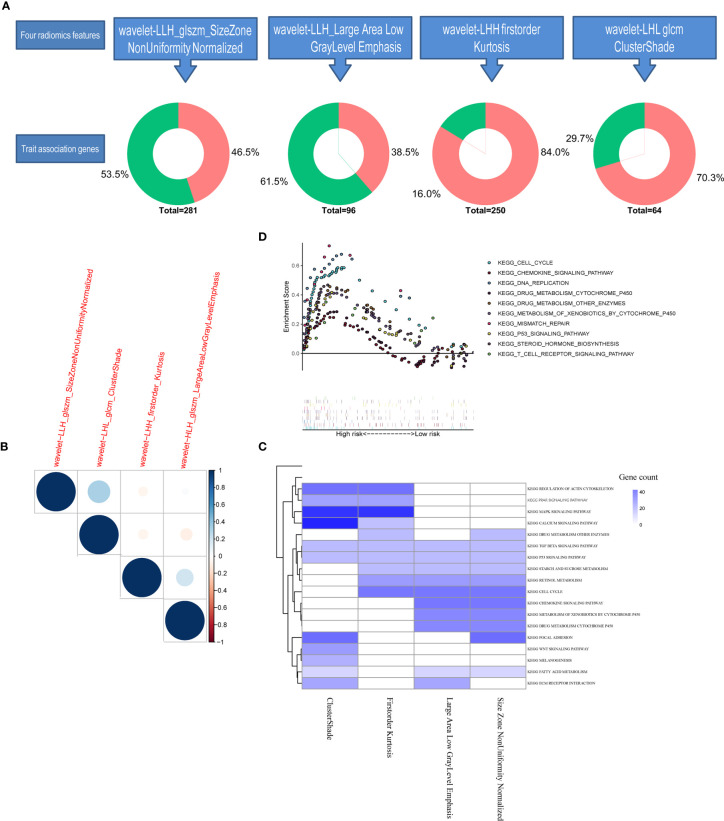
Tarit-association genes and functional enrichment analysis. **(A)** Each differently expressed gene in four radiomics features in the genomic study. **(B)** Heatmap of the similarity between each train-associated gene calculated by the Spearman index. **(C)** Heatmap of GESA enrichment analysis. **(D)** GSEA enrichment analysis of the radiomics score model.

We further conducted genomic analysis to explore the molecular underpinning of the identified all-relevant features by evaluating possible radiogenomics links using the RNA-Sep technology. More than 70% of significantly different expression genes in the module of glszm Size Zone NonUniformity Normalized and glcm Cluster Shade were upregulated in the tumor tissues, while a small part of genes were negatively expressed (**Figure 6A**). When we examined the degree of overlap between radiomics feature genes to investigate the dependence between each feature, glcm Cluster Shade and glszm Large Area Low Gray Level Emphasis showed an overall high similarity (**Figure 6B**). The pre-ranked GSEA showed that the significant enriched pathways (FDR <0.1) among the top associations with these four radiomics factors were mostly correlated with drug metabolism and chemokine regulation (**Figure 6C**). Of the radiomics score signatures, the most enriched pathways were also gathered in the drug metabolism cytochrome P450 and other enzymes (**Figure 6D**). These results revealed that the developed imaging biomarker might reflect the different drug metabolic changes during cancer therapy, which could better stratify patients for more precise therapeutic care.

## Discussion

In consideration of the complexity of the heterogeneity of the morphologic, biologic, and clinical nature, conventional classification systems were no longer able to reflect the complex molecular nature of GC. As for proposed morphology-based classification systems, the World Health Organization (WHO) (papillary, tubular, mucinous, and poorly cohesive (PCC-NOS)/signet ring) (16) and the Lauren (intestinal, diffuse, and mixed) classifications are the most commonly preferred (17). As for comprehensive genomic assays, the TCGA Consortium classified GC into four major genomic subtypes, including tumors positive for Epstein–Barr virus (EBV), microsatellite unstable tumors (MSI), genomically stable tumors (GS), and tumors with chromosomal instability (CIN) (18); the Asian Cancer Research Group (ACRG) divided GC into four subtypes: MSI, microsatellite stable (MSS)/epithelial–mesenchymal transition (EMT), MSS/tumor protein 53 (TP53) active, and MSS/TP53 inactive (19). Moreover, a plethora of genomic alterations have been identified, such as HER2, FGFR2, EGFR, PI3K, mTOR, and MET, which provided good identification of GC patients who derive therapeutic benefit from ACT (20). Therefore, the existence of GC intra-tumor heterogeneity affects the accuracy of clinical decisions and lead to a substantial discordance rate. Thus, researchers are now investigating auxiliary noninvasive approaches to precisely predict the therapy of GC preoperatively.

Radiomics is a promising tool which defines mathematical features from medical images using a series of data-characterization algorithms. This technique allows doctors to access standardized image texture information and to stand out informed inference. To date, radiomics has been proven to effectively predict biological characteristics of numerous types of cancers (7, 19, 20). In GC patients, although studies were preliminary, radiomics texture analyses have been proven to not only improve prediction of survival but also provide additional information in oncologic practice related to benign and malignant nodule differentiation, prediction of lymph node metastasis, histological subtype classification, response to chemotherapy assessment, and mutation type identification (9, 15, 17). In this study, a total of 799 two-dimensional features were extracted from each ROI by PyRadiomics. After dispelling redundancies, four selected radiomics features were extracted to construct a radiomics signature, which enabled more accurate identification of GC patients who might benefit from postoperation chemotherapy. As shown in the present study, the radiomics signature successfully identified high-risk GC patients with poor survival outcomes, for whom more intensified treatment was needed. Furthermore, for patients with a low radiomics score were more inclined to fall victim in the postoperation chemotherapy. By adding the clinical features, we also constructed the clinical-radiomics feature nomogram for predicting survival of GC after gastrectomy.

Surgical resection is the main curative method for GC, but the high rate of relapse in patients makes it important to consider adjuvant treatment selection. Current guidelines have strongly proven chemotherapy as a standard component for advanced GC therapies, whereas existing studies provided that a subgroup of patients does not benefit from the present ACT. At present, three major international GC guidelines guide the population indicated for ACT, which can be roughly divided into Europe (European Society for Medical Oncology, ESMO), United States (National Comprehensive Cancer Network, NCCN), and East Asia (21). Based on the MAGIC trial, ESMO recommended ACT for stage >T1N0 (22) and NCCN suggested clinical stage ≥ T2; yet, the Japanese guidelines still suggested all surgeries combined with postoperative chemotherapy (23). Although the efficacy of ACT for GC has been proven, there is no ideal measure for reasonable noninvasive selection, especially for early postoperative GC patients. The NCCN guidelines recommend ACT for pT1N1 gastric cancer patients after curative resection (24); on the contrary, the Japanese Gastric Cancer Treatment Guidelines did not show any ACT benefit with regard to pT1N1 gastric cancer patients after curative resection (24). By and large, the risk factors associated with postoperation chemotherapy selection include tumor invasion, lymph node metastases, tumor stage and Borrmann type, dMMR, gene mutant, family history, and physical condition. Although intensive protocols are promising, selecting the optimal adjuvant chemotherapy remains a difficult task that requires a balance between the therapeutic benefits and toxicity. The most common adverse events were asthenia/anorexia (33.3%), hematologic malignancies (29.6%), and infection (14.8%) (25). Thus, a biomarker study is urgently necessary for selecting the GC subgroups for which adjuvant treatment provides an oncological benefit postoperation. Our study provided a statistically robust approach to construct the radiomics signature for the administration of ACT in GC. The radiomics signature provides the incremental value for guiding the adoption of ACT in patients with a low radiomics score. The radiomics score elucidated the relationship between tumor characteristics and their imaging appearance as well as developed imaging biomarkers that can predict risk and outcomes, thereby better stratifying patients for more precise therapeutic care.

The radiogenomics analysis provided that a prognostic radiomics signature could capture tumor cell intratumor heterogeneity, which is also associated with underlying gene expression patterns. The radiogenomics analysis showed multiple associations between CT image features and trait-associated genes mostly correlated with various drug metabolisms and chemokine regulation. Given that radiomics signature provided the incremental value for the adoption of ACT, although the mechanism of the relationship between radiomics features and chemotherapy has not been shown thoroughly, we speculated that it may be associated with the strong correlation with cell cycling pathways, chemokine signaling, and chemotherapeutic drug metabolism (**Figure 6**). The present image-to-molecular feature associations could also be applied to assess therapeutic options based on biological pathway activity. The effects of whole-body chemotherapy for GC may be deeply influenced by drug metabolism and critical signaling pathways. First-Order Kurtosis, Large Area Low Gray Level Emphasis, and Size Zone NonUniformity Normalized could be effectively targeted by drug metabolism *via* cytochrome P450 and other enzymes, retinol metabolism, and sucrose metabolism. The gene pathway analysis indicated that radiogenomics may be suitable for predicting the efficacy of pathway–target therapies.

However, some limitations in this study should also be noticed. Firstly, the small sample size of GC patients and the retrospective nature of the data collection possibly affect the statistical power. We need to increase the sample size and conduct a multicenter research to verify the accuracy and stability of the radiomics nomogram model. Secondly, the decision to treat or not to treat patients after surgery was made by the patients and/or clinicians, and the use of adjuvant chemotherapy was not within a randomized comparison. Chemotherapy drug side effects, types of chemotherapy selection, and irregular course of chemotherapy all existed in our follow-up study, which inevitably affected the outcome. Moreover, radiomics does indeed suffer from a closed-source nature, unharmonized acquisition settings, discordant reconstruction parameters, lack of interpretability, redundancy, and methodological bias. Complementary innovations in genetic and imaging-based studies that allow for the spatial quantification of tumor heterogeneity could provide a realization of precision oncology.

## Data Availability Statement

The original contributions presented in the study are included in the article/**Supplementary Material**. Further inquiries can be directed to the corresponding authors.

## Ethics Statement

Written informed consent was obtained from the individual(s) for the publication of any potentially identifiable images or data included in this article.

## Author Contributions

WC and YJ conceived of and designed the experiments. YL, YX, and RC performed the data collection. WC and YX analyzed the data. WC, YX, and YJ wrote the manuscript. All authors contributed to the article and approved the submitted version.

## Funding

This work was partially supported by grants from the Natural Science Foundation of Zhejiang Province of China (LY18H030008); Wu Jieping Medical Foundation (320.6750.17396), Wenzhou Science and Technology Bureau (No. 20190432), and the First Affiliated Hospital of Wenzhou Medical University (Grant No. FHY2019002).

## Conflict of Interest

The authors declare that the research was conducted in the absence of any commercial or financial relationships that could be construed as a potential conflict of interest.

## Publisher’s Note

All claims expressed in this article are solely those of the authors and do not necessarily represent those of their affiliated organizations, or those of the publisher, the editors and the reviewers. Any product that may be evaluated in this article, or claim that may be made by its manufacturer, is not guaranteed or endorsed by the publisher.

## Supplementary Material

Supplementary Figure 1 Forest plot showing the logistic regression analyses of the four radiomic features.

Supplementary Figure 2 The distribution of radiomics score and disease-free survival. **(A)** The distribution of radiomics score of GC patients; **(B)** The disease-free survival status for each GC patients; **(C)** disease-free survival curve of total GC patients; **(D)** PCA depicts the variation of high- and low- radiomics score; **(E)** disease-free survival and ROC curves of training group; **(F)** disease-free survival and ROC curves of validation group.

Supplementary Figure 3 Validation analysis of the radiomics score in TCGA cohort. **(A)** Overall survival curve of the TCGA radiomics score. **(A)** ROC curves of TCGA radiomics score.

Supplementary Figure 4 The prediction performance analysis of disease-free survival. **(A)** The nomogram for predicting 1-, 3- and 5-year PFS after surgery; **(B)** Calibration curve for risk of 1- year disease-free survival; **(C)** Calibration curve for risk of 3-year disease-free survival; **(D)** Calibration curve for risk of 5-year disease-free survival; **(E)** Receiver operating characteristic (ROC) curves for 1-, 3- and 5- year disease-free survival.
