# Supplementary figures and images for: Integrative Radiogenomics Approach for Risk Assessment of Postoperative and Adjuvant Chemotherapy Benefits for Gastric Cancer Patients
Source: Front Oncol. 2021 Nov 5;11:755271. doi: 10.3389/fonc.2021.755271 (PMC8602567; doi:10.3389/fonc.2021.755271)

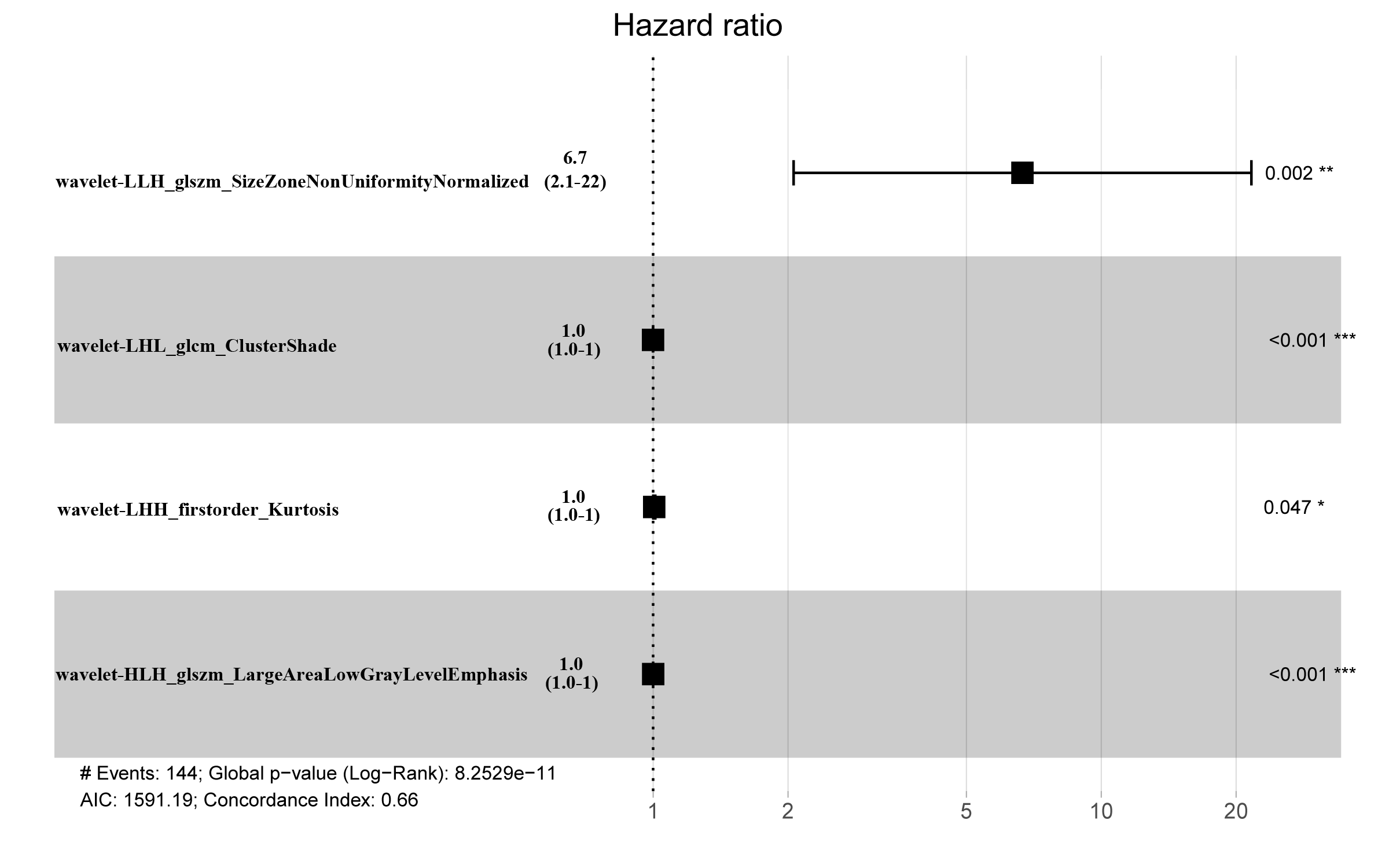

Supplement: Supplementary Figure 1 — Forest plot showing the logistic regression analyses of the four radiomic features. [file Image_1.tif]

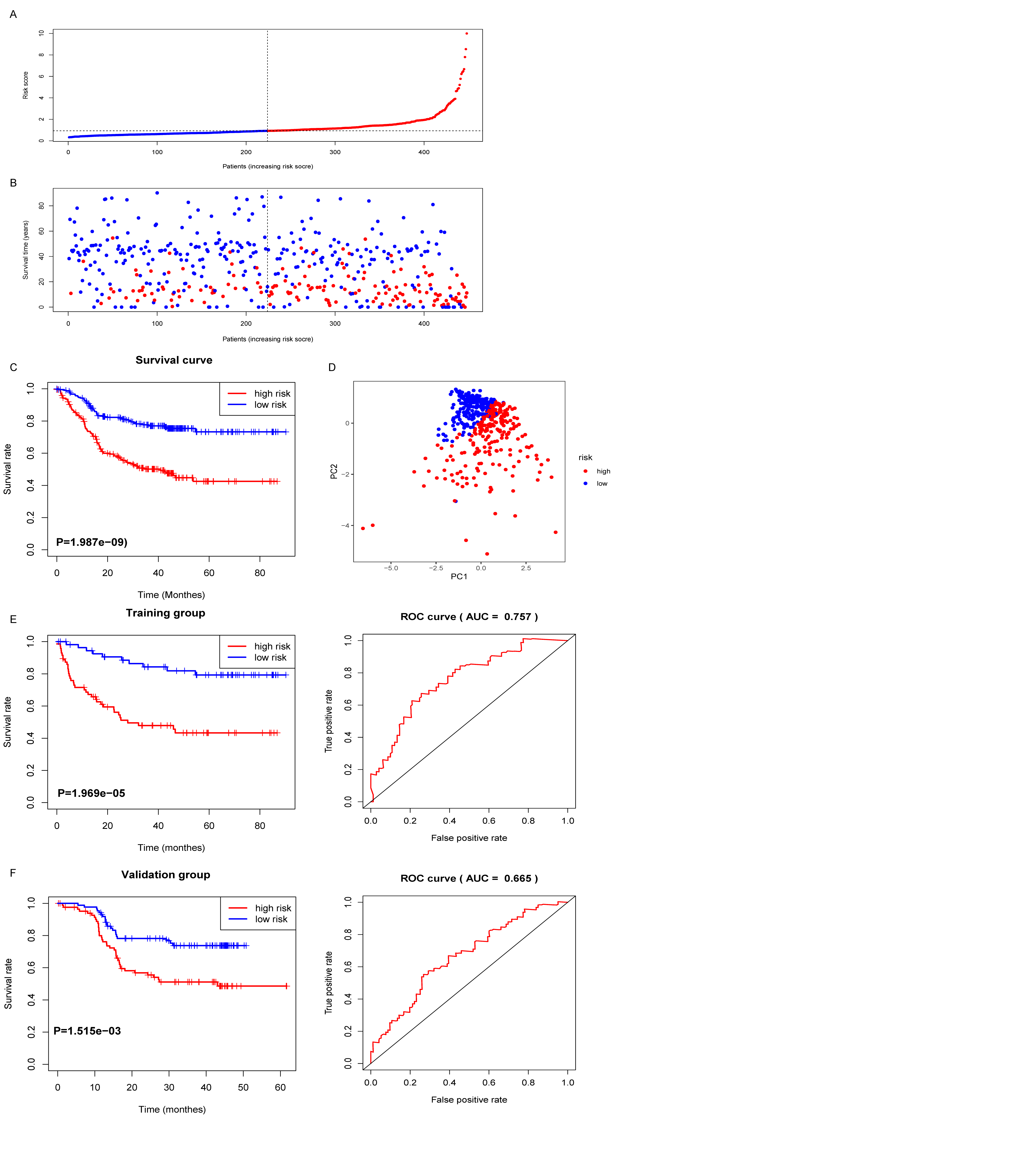

Supplement: Supplementary Figure 2 — The distribution of radiomics score and disease-free survival. (A) The distribution of radiomics score of GC patients; (B) The disease-free survival status for each GC patients; (C) disease-free survival curve of total GC patients; (D) PCA depicts the variation of high- and low- radiomics score; (E) disease-free survival and ROC curves of training group; (F) disease-free survival and ROC curves of validation group. [file Image_2.tif]

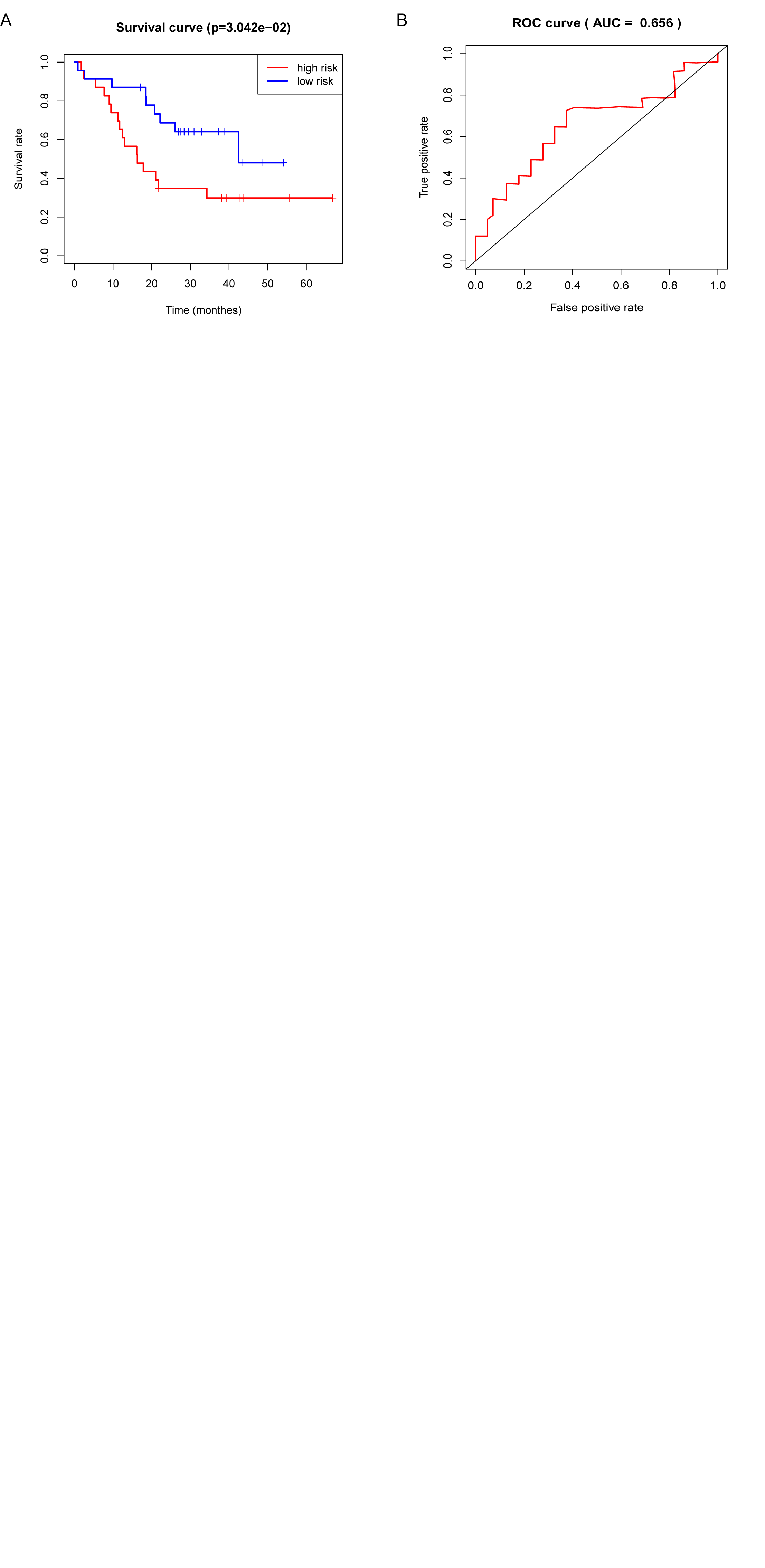

Supplement: Supplementary Figure 3 — Validation analysis of the radiomics score in TCGA cohort. (A) Overall survival curve of the TCGA radiomics score. (A) ROC curves of TCGA radiomics score. [file Image_3.tif]

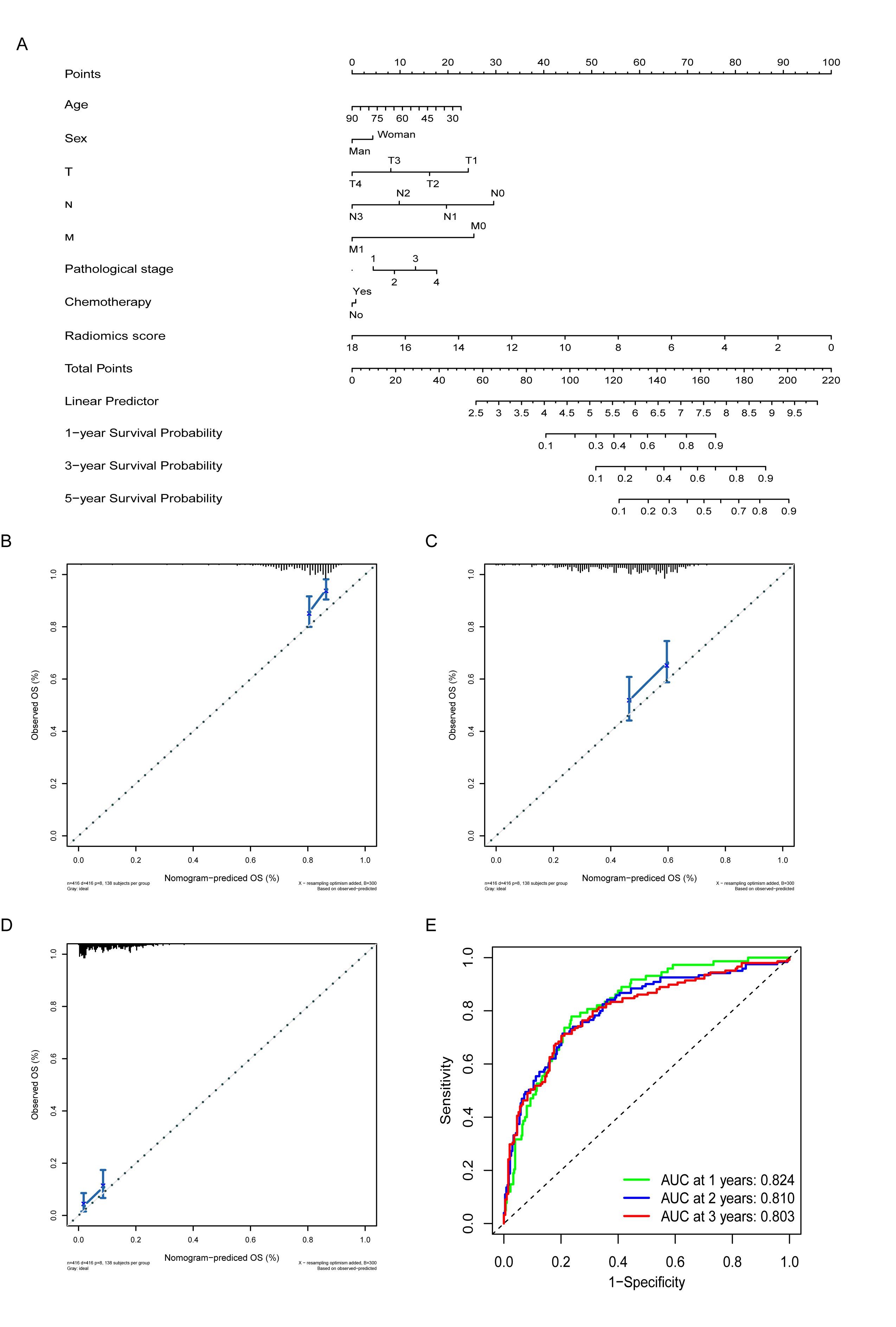

Supplement: Supplementary Figure 4 — The prediction performance analysis of disease-free survival. (A) The nomogram for predicting 1-, 3- and 5-year PFS after surgery; (B) Calibration curve for risk of 1- year disease-free survival; (C) Calibration curve for risk of 3-year disease-free survival; (D) Calibration curve for risk of 5-year disease-free survival; (E) Receiver operating characteristic (ROC) curves for 1-, 3- and 5- year disease-free survival. [file Image_4.tif]
