# Supplementary material for: Integrative Radiogenomics Approach for Risk Assessment of Postoperative and Adjuvant Chemotherapy Benefits for Gastric Cancer Patients
Source: Front Oncol. 2021 Nov 5;11:755271. doi: 10.3389/fonc.2021.755271 (PMC8602567; doi:10.3389/fonc.2021.755271)
Supplement: Supplementary file 5 [file Table_1.docx]

sTable 1 The baseline characteristics of GC patients with in the training and validation, external

| characteristic | Total | Training cohort | Validation cohort | TCGA GC |
| --- | --- | --- | --- | --- |
| Age, year | 417 | 172 | 245 | 41 |
| <65 | 193(47.2%) | 84(48.8%) | 106(43.3%) | 16(39.0%) |
| ≥65 | 224(52.8%) | 88(51.2%) | 139(56.7%) | 25(61.0%) |
| Gender |  |  |  |  |
| Man | 142(34.1%) | 90(52.3%) | 52(21.2%) | 35(85.4%) |
| Woman | 275(65.9%) | 82(47.7%) | 193(78.8%) | 6(14.6%) |
| T stage |  |  |  |  |
| T1-2 | 140(34.1%) | 54(31.4%) | 86(35.1%) | 2(4.9%) |
| T3-4 | 277(65.9%) | 118(68.6%) | 159(64.8%) | 39(95.1%) |
| N stage |  |  |  |  |
| N0 | 179(42.9%) | 81(47.1%) | 98(40.0%) | 10(24.4%) |
| N1-3 | 338(57.1%) | 91(52.9%) | 147(60.0%) | 31(75.6%) |
| M stage |  |  |  |  |
| M0 | 384(92.1%) | 162(94.2%) | 210(85.7%) | 40(97.6%) |
| M1 | 33(7.9%) | 10(5.8%) | 23(14.3%) | 1(2.4%) |
| Pathological stage |  |  |  |  |
| 1-2 | 172(36.5%) | 74(43.0%) | 98(40.0%) | 9(22.0) |
| 3-4 | 245(63.5%) | 98(57.0%) | 147(60.0%) | 32(78.0) |
| Chemotherapy |  |  |  |  |
| No | 182(43.6%) | 85(49.4%) | 97(36.6%) | / |
| Yes | 235(56.4%) | 87(50.6%) | 148(63.4%) | / |
| Follow-up  Time, Month |  |  |  |  |
|  | 32.26 | 30.25 | 31.42 | 25.37 |

validation cohorts
